# Multi-Vehicle Cooperative Target Tracking with Time-Varying Localization Uncertainty via Recursive Variational Bayesian Inference

**DOI:** 10.3390/s20226487

**Published:** 2020-11-13

**Authors:** Xiaobo Chen, Yanjun Wang, Ling Chen, Jianyu Ji

**Affiliations:** 1 Automotive Engineering Research Institute, Jiangsu University, Zhenjiang 212013, China; 2 School of Automotive and Traffic Engineering, Jiangsu University, Zhenjiang 212013, China; 18651403397@163.com (Y.W.); lingchenxue3@163.com (L.C.); 18852852561@163.com (J.J.)

**Keywords:** target tracking, cooperative perception, variational Bayesian inference, joint state estimation

## Abstract

Cooperative target tracking by multiple vehicles connected through inter-vehicle communication is a promising way to improve the estimation of target state. The effectiveness of cooperative tracking closely depends on the accuracy of relative localization between host and cooperative vehicles. However, the localization signal usually provided by the satellite-based navigation system is rather susceptible to dynamic driving environment, thus influencing the effectiveness of cooperative tracking. In order to implement reliable cooperative tracking, especially when the statistical characteristic of the relative localization noise is time-varying and uncertain, this paper presents a recursive Bayesian framework which jointly estimates the state of the target and the cooperative vehicle as well as the localization noise parameter. An online variational Bayesian inference algorithm is further developed to achieve efficient recursive estimate. The simulation results verify that our proposed algorithm can effectively boost the accuracy of target tracking when the localization noise dynamically changes over time.

## 1. Introduction

Intelligent vehicles (IVs) [1] equipped with different types of on-board sensors, such as Lidar, Radar, Camera, etc, can collect the information about surrounding environment. By analyzing this information, the IVs can achieve reliable situational awareness [2] and thus make correct decisions [3,4]. Among various environment perception tasks, target tracking [5,6] plays a crucial role for many autonomous driving functions, such as the forward collision assistance or adaptive cruise control. Traditionally, the state of target is estimated based on the data collected by on-board sensors. Consequently, the tracking performance is restricted due to the limitation of sensors. For example, the mutual occlusion between targets will prevent the IVs to recognize those occluded targets [7]. Recently, due to the fast development of inter-vehicle and cellular communication, cooperative perception through vehicle-to-vehicle (V2V) or vehicle-to-infrastructure (V2I) communication has attracted much attention [4,7]. The IVs equipped with dedicated short range communication (DSRC) radios [8] or 4G/5G technology [9] are able to exchange their information including the position, speed, heading, etc., with other neighboring IVs or roadside units (RSUs). Then, the vehicle receiving such information can fuse the transmitted data with its local information to improve the state estimate of target and extend the perception range. In the foreseeable future, a standardized 5G network can enable vehicular communication with remarkably low power consumption, high peak data rate, and low latency [10].

During the last few years, many studies have been proposed to fuse the information from multiple sources in cooperative intelligent transportation systems (C-ITS) areas [11]. In this study, we focus on cooperative target tracking via multiple vehicles, although the developed algorithm is also applicable for other cooperative situations. For convenience, we call the vehicle transmitting information as the cooperative vehicle (CV), while the vehicle receiving information as the host vehicle (HV). Depending on the information shared among vehicles, the architecture of cooperative perception can be categorized into three types: data-level, feature-level, and track-level fusion.

For data-level fusion [12], CV directly forwards the raw measurements, such as point cloud or image, to HV, where most processing operations, including spatial registration and state estimation, are carried out. For feature-level fusion [13], on the other hand, the raw data are partially processed independently by each vehicle so as to extract low-dimensional features, which are then exchanged among vehicles. Different from the above two types of fusion, track-level fusion supposes that the raw measurements are completely processed by each vehicle to obtain local state estimation of the target. Then, the local estimation is transmitted to other vehicles for further fusion. Generally, data-level fusion can yield more accurate estimation because it can make full use of the available information. However, it may cause large computation and communication burden. In contrast, track-level fusion [14,15] reduces the communication bandwidth; however, may lose useful information. Feature-level fusion strikes the balance between computation and communication load, and, hence, can be viewed as compromise between data-level and track-level fusion.

In [16], a cooperative perception approach exchanging raw LiDAR data among vehicles was proposed. In [13], an algorithm which can share the feature extracted from point cloud data among multiple vehicles was developed. In [17], a collaborative tracking algorithm was proposed for the tracking of multiple vehicles. PHD filter [18] was independently executed by HV and CV to obtain the state of vehicles. The PHD density was then transformed from the coordinate frame of CV to that of HV and then merged with local estimate via covariance intersection (CI) fusion [19]. The resulting cooperative perception was further applied for the overtaking decision [20].

In order to achieve reliable cooperative tracking, the relative localization between HV and CV is an important factor used to convert the local measurement or track estimation of target from the frame of one vehicle to that of another. In the literatures, most works assume such information is perfectly known when conducting cooperative tracking [21]. Currently, satellite-based navigation system, such as global positioning system (GPS) and Beidou, has been widely applied in the localization of vehicles. However, the localization signal is subject to noise from different sources. The signal can be degraded or even blocked when traveling in dense urban areas close to building or vegetation, thus leading to large localization errors [22,23]. As a result, the relative location between vehicles calculated from the localization signal is expected to be uncertain and time-varying. Few works investigated the cooperative tracking problem with imperfect localization information. In [24], the high-precision and low-precision positioning situations are, respectively, characterized by two observation likelihood models. In [25], an approach for jointly estimating the state of vehicle and targets based on Poisson multi-Bernoulli filter (PMBM) [26] was developed to consider the uncertain localization of vehicle. However, the localization noise covariance was assumed to be exactly known. In [27], a cooperative pedestrian tracking algorithm was presented in the GPS-denied environments. In [28], a robust cooperative tracking algorithm was proposed for the situation where the localization information is not available.

In summary, achieving reliable cooperative target tracking especially when the relative localization noise is uncertain and time-varying is a nontrivial task. Therefore, this paper presents a recursive Bayesian framework to address the above problem. Specifically, both HV and CV collect the measurement of the same target while the information collected by CV is transmitted to HV. Under the assumption that the uncertainty of relative position and orientation between two vehicles may vary over time, HV attempts to estimate the joint state of target and CV as well as the noise parameter based on the measurements. To guarantee the conjugate relationship, a solution algorithm based on variational Bayesian (VB) inference is then developed, such that the system state and the noise parameter can be alternatively corrected. VB inference is widely applied in machine learning community [29,30] and has been successfully introduced into target tracking and sensor fusion [31,32,33] in recent years. Overall, this paper is interesting from the following aspects

1. Our algorithm can be used in dynamic environments, in the sense that the statistical characteristic of the localization noise is uncertain and time-varying.
2. The cooperative tracking problem is formulated in recursive Bayesian framework and an efficient VB inference algorithm is proposed.
3. Simulation shows that the tracking error of cooperative tracking can be reduced by 18.15% to 9.5% compared with non-cooperative tracking, depending on the uncertainty level of the localization noise.

The remainder of this paper is organized as follows. In Section 2, we give the system description and model definition based on recursive Bayesian framework. In Section 3, the principle of VB inference is briefly explained. Then, the specific solution algorithm for the estimation of system state and the localization noise parameter is presented in Section 4. In Section 5, the proposed approach is evaluated through computer simulation experiments. Finally, Section 6 gives the conclusions of the paper.

## 2. System Description and Model Definition

**Figure 1:**
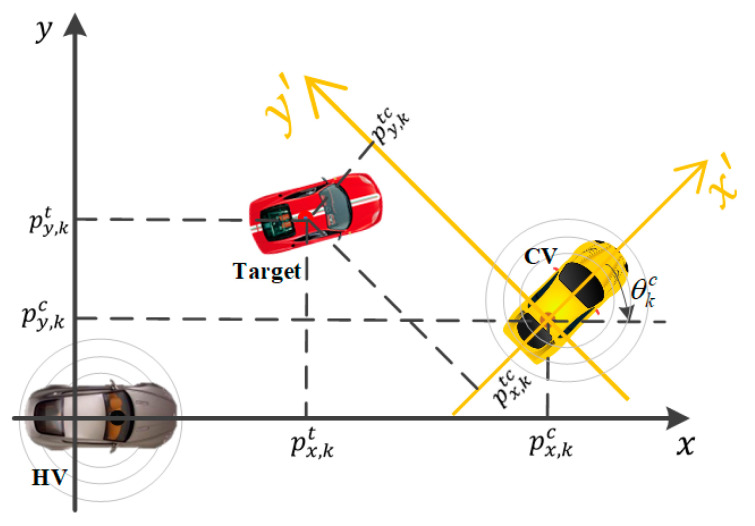
Schematic diagram of cooperative tracking scene (HV: host vehicle, CV: cooperative vehicle).

The scenario considered in this work is shown in Figure 1. The host and cooperative vehicles collect the information about the same target based on their individual on-board sensors. Then, CV transmits the measurement about the target along with its own localization information to HV. Finally, HV fuses the received measurement with its own measurement about the same target based on the relative localization between HV and CV. It should be emphasized that the scenario considered in this work is very universe and can be extended to more complex situations through integrating with other technologies. For example, by introducing data association, such as global nearest-neighbor method, etc., our model can be immediately used for multiple target tracking.

As shown in Figure 1, taking the coordinate frame of HV as reference frame, we assume that the motion state of both target and CV evolve following linear state space model. Therefore, we can combine their individual motion state together to yield an augmented dynamic model as

(1) $$x_{k}=F_{k}x_{k-1}+w_{k},$$

where  k=1,2,3,⋯  is the time step, $x_{k}\in R^{n}\;$ is the system state. Specifically, $x_{k}={\left[x_{k}^{t},x_{k}^{c}\right]}^{T}$ consists of two parts: $x_{k}^{t}={\left[p_{x,k}^{t},p_{y,k}^{t},{\dot{p}}_{x,k}^{t},{\dot{p}}_{y,k}^{t}\right]}^{T}$, representing the motion state of the target at time step *k*, including the position and velocity along x and y direction, $x_{k}^{c}={\left[p_{x,k}^{c},p_{y,k}^{c},{\dot{p}}_{x,k}^{c},{\dot{p}}_{y,k}^{c},\theta_{k}^{c},{\dot{\theta }}_{k}^{c}\right]}^{T},\;$ representing the motion state of the cooperative vehicle, including extra heading angle $\theta_{k}^{c}$ and heading angle speed ${\dot{\theta }}_{k}^{c}$ besides the position and velocity as the target. $F_{k}\in R^{n\times n}\;$ is the state transition matrix, $w_{k}\in R^{n}\;$ is the process noise following Gaussian distribution $N\left(w_{k};0,Q\right)$ with zero mean and covariance matrix Q. We also assume that the system state $x_{1}$ at the first time step is subject to have a Gaussian distribution with mean vector ${\hat{x}}_{1|1}$ and the covariance matrix $P_{1|1}$, i.e., $N\left(x_{1};{\hat{x}}_{1|1},P_{1|1}\right)$.

For the cooperative tracking scenario we consider in this work, the statistical property of the relative localization noise of cooperative vehicle is time-varying and uncertain while the statistical property of observation noise of target by on-board sensor of HV and CV is known and stable. Therefore, we have the following measurement equation of the system state

(2) $$\{\begin{array}{c} y_{k}^{1}=h\left(x_{k}\right)+v_{k}^{1}\\ y_{k}^{2}=Hx_{k}+v_{k}^{2}\; \end{array},$$

where $y_{k}^{1}={\left[y_{k}^{t},y_{k}^{tc}\right]}^{T}\in R^{m_{1}}$, $y_{k}^{2}={\left[y_{k}^{c},\theta_{k}^{c}\right]}^{T}\in R^{m_{2}}$. Specifically, $y_{k}^{t}\;$ and $y_{k}^{tc}$ represent the measurement of the same target in the coordinate frame of HV and CV, respectively. $v_{k}^{1}$ is the corresponding measurement noise. $y_{k}^{c}$ and $\theta_{k}^{c}$ are the measurements of CV in the coordinate frame of HV, including the observed position in x and y direction and heading angle. In this work, we assume that the measurement noise $v_{k}^{1}$ obeys Gaussian distribution $N(v_{k}^{1};0,\Sigma_{k}^{1})$ with known covariance matrix $\Sigma_{k}^{1}\in R^{m_{1}\times m_{1}}$ while the measurement noise $v_{k}^{2}$ also follows Gaussian distribution $N(v_{k}^{2};0,\Sigma_{k}^{2})$; however, with uncertain and time-varying covariance matrix $\Sigma_{k}^{2}=\mathrm{diag}(\sigma_{k|k,1}^{2}\ldots ,\sigma_{k|k,m_{2}1}^{2})$, with diag(·) denoting a diagonal matrix.

According to the relationship between the coordinate frames of HV and CV shown in Figure 1, the measurement equation $h\left(x_{k}\right)$ in (2) can be expressed as

(3) $$h\left(x_{k}\right)=\left[\begin{array}{ccccc} \begin{array}{cc} 1 & 0\\ 0 & 1 \end{array} & \begin{array}{cc} 0 & 0\\ 0 & 0 \end{array} & \begin{array}{cc} 0 & 0\\ 0 & 0 \end{array} & \begin{array}{cc} 0 & 0\\ 0 & 0 \end{array} & \begin{array}{cc} 0 & 0\\ 0 & 0 \end{array}\\ R & \begin{array}{cc} 0 & 0\\ 0 & 0 \end{array} & -R & \begin{array}{cc} 0 & 0\\ 0 & 0 \end{array} & \begin{array}{cc} 0 & 0\\ 0 & 0 \end{array} \end{array}\right]x_{k},$$

where $R=\left[\begin{array}{cc} cos\theta_{k}^{c} & sin\theta_{k}^{c}\\ -sin\theta_{k}^{c} & cos\theta_{k}^{c} \end{array}\right]$. For the observation $y_{k}^{2}$, we have the measurement matrix in (2) as

(4) $$H=\left[\begin{array}{ccccc} \begin{array}{cc} 0 & 0\\ 0 & 0 \end{array} & \begin{array}{cc} 0 & 0\\ 0 & 0 \end{array} & \begin{array}{cc} 1 & 0\\ 0 & 1 \end{array} & \begin{array}{cc} 0 & 0\\ 0 & 0 \end{array} & \begin{array}{cc} 0 & 0\\ 0 & 0 \end{array}\\ \begin{array}{cc} 0 & 0 \end{array} & \begin{array}{cc} 0 & 0 \end{array} & \begin{array}{cc} 0 & 0 \end{array} & \begin{array}{cc} 0 & 0 \end{array} & \begin{array}{cc} 1 & 0 \end{array} \end{array}\right],$$

In our online estimation model, the measurement set $y_{1:k}=\left\{y_{1},\ldots ,y_{k}\right\}$ is observable, while the system state $x_{k}$ and the localization noise covariance $\Sigma_{k}^{2}$, are viewed as hidden variables and need to be estimated based on $y_{1:k}$. The aim of optimal recursive Bayesian filtering is to estimate the posterior probability distribution of $\;x_{k}\;$ and ${\;\Sigma }_{k}^{2}\;$ based on the observed data $y_{1:k}$, so as to realize the joint estimation of the unknown variables. It generally consists of two steps. First, according to the Chapman–Kolmogorov (CK) equation, the prediction of the unknown variables is given by

(5) $$p\left(x_{k},\Sigma_{k}^{2}|y_{1:k-1}\right)=\int p\left(x_{k},\Sigma_{k}^{2}|x_{k-1},\Sigma_{k-1}^{2}\right)\times p\left(x_{k-1},\Sigma_{k-1}^{2}|y_{1:k-1}\right)dx_{k-1}d\Sigma_{k-1}^{2},$$

Then, the observed information is incorporated by the well-known Bayesian theorem, thus yielding the following correction equation

(6) $$p\left(x_{k},\Sigma_{k}^{2}|y_{1:k}\right)=\frac{p\left(y_{k}|x_{k},\Sigma_{k}^{2}\right)p\left(x_{k},\Sigma_{k}^{2}|y_{1:k-1}\right)}{\int p\left(y_{k}|x_{k},\Sigma_{k}^{2}\right)p\left(x_{k},\Sigma_{k}^{2}|y_{1:k-1}\right)dx_{k}d\Sigma_{k}^{2}},$$

Given the measurements $y_{1:k-1}$, we assume the joint posterior distribution of $x_{k-1}$ and ${\;\Sigma }_{k-1}^{2}$ at time step k−1 can be approximated as the product of the Gaussian distribution and the inverse-Gamma distribution, that is

(7) $$p\left(x_{k-1},\Sigma_{k-1}^{2}|y_{1:k-1}\right)=p\left(x_{k-1}|y_{1:k-1}\right)p\left(\Sigma_{k-1}^{2}|y_{1:k-1}\right),$$

where we have

(8) $$\{\begin{array}{c} p\left(x_{k-1}|y_{1:k-1}\right)=N\left(x_{k-1};{\hat{x}}_{k-1|k-1},P_{k-1|k-1}\right)\;\;\;\;\\ p\left(\Sigma_{k-1}^{2}|y_{1:k-1}\right)=\prod_{t=1}^{m_{2}}IG\left(\sigma_{k-1,t}^{2};\alpha_{k-1|k-1,t},\beta_{k-1|k-1,t}\right) \end{array},$$

**Figure 2:**
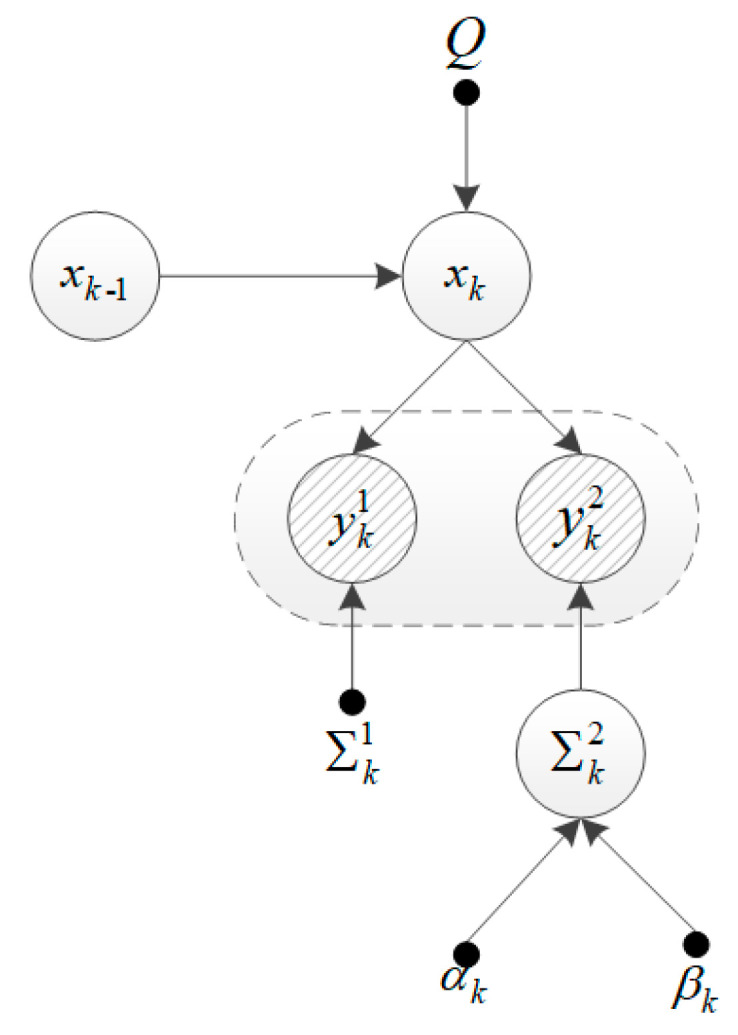
Probabilistic graphical model representation of our model.

Here, $IG\left(\sigma_{k-1,t}^{2};\alpha_{k-1|k-1,t},\beta_{k-1|k-1,t}\right)$ denotes the inverse-Gamma distribution with the scale parameter $\;\alpha_{k-1|k-1,t}\;$ and the shape parameter $\beta_{k-1|k-1,t}$. In summary, the proposed cooperative tracking model under uncertain localization noise can be represented as the probabilistic graphical model (PGM) [34] shown in Figure 2.

## 3. Principle of Variational Bayesian Inference

Suppose that we want to infer the posterior distribution p(ϕ|Z) of the hidden variable ϕ given the observed data Z under the Bayesian framework. Based on the Bayes’ theorem, we have

(9) $$p\left(\phi |Z\right)=\frac{p\left(Z|\phi \right)p\left(\phi \right)}{p\left(Z\right)}=\frac{p\left(Z|\phi \right)p\left(\phi \right)}{\int p\left(Z|\phi \right)p\left(\phi \right)d\phi },$$

where  p(Z|ϕ) is the likelihood of observed data Z, p(ϕ) is the prior distribution and p(Z) denotes the marginal likelihood of data. For many problems, it is infeasible to evaluate the posterior p(ϕ|Z)  analytically or numerically. To address this problem, VB inference was proposed to find variational distribution of hidden variables that can approximate true posterior distribution as closely as possible. Specifically, we always have the following equation for the logarithm of p(Z) [35]

(10) logp(Z)=F(q(Φ))+KL(q(Φ)||p(ϕ|Z)),

where q(Φ) is the introduced variational distribution aiming to approximate the intractable posterior distribution p(ϕ|Z); F(q(Φ)) is the free energy of the following form

(11) $$F\left(q\left(\Phi \right)\right)=\int q\left(\Phi \right)\log\frac{p\left(\Phi ,Z\right)}{p\left(Z\right)}d\Phi ,$$

and KL(q(Φ)||p(ϕ|Z)) denotes the Kullback–Leibler (KL) divergence between q(Φ) and  p(ϕ|Z), which is used to measure the consistency between two probability distributions. That is,

(12) $$KL\left(q\left(\Phi \right)||p\left(\phi |Z\right)\right)=\int q\left(\Phi \right)\log\frac{q\left(\Phi \right)}{p\left(\Phi |Z\right)}d\Phi ,$$

Equation (10) indicates that the sum of free energy and KL divergence is always equal to the logarithm of marginal likelihood. In VB framework, we seek for the variational posterior q(Φ) by minimizing KL divergence (12), indicating that the variational distribution is able to approximate the true posterior. However, it is infeasible to minimize (12) directly because p(Φ|Z) is unknown. To avoid this issue, we can instead maximize the free energy F(q(Φ)) in (11), since logp(Z) is constant given observation Z. Furthermore, let Φ can be partitioned into disjoint groups $\Phi_{1},\Phi_{2},\cdots ,\Phi_{M}$ where ${⋃}_{m=1}^{M}\Phi_{m}=\Phi$. Then, based on the mean field theory, we suppose that  q(Φ)  has a factorized form as

(13) $$q\left(\Phi \right)=\prod_{m=1}^{M}q\left(\Phi_{m}\right),$$

It indicates that $\Phi_{m}$ and $\Phi_{l}$ (l≠m) are independent to each other. In order to maximize F(q(Φ)) with factorized form (13), an iterative approach is adopted where each $q\left(\Phi_{m}\right)$ is alternatively optimized while keeping the other $q\left(\Phi_{l}\right)$ fixed, l≠m. In such a case, the optimal $q\left(\Phi_{m}\right)$ is given by [35]

(14) $$\log q\left(\Phi_{m}\right)\propto {\langle \log p(\Phi ,Z)\rangle }_{q\left(\Phi_{l}\right),l\neq m},$$

where ${\langle \cdot \rangle }_{q\left(\Phi_{l}\right),l\neq m}$ is the expectation with respect to all $\Phi_{l}$ except $\Phi_{m}$. The above iteration continues until some convergence criterion is satisfied.

## 4. Online Variational Bayesian Inference of Parameters

In this section, we concentrate on the solution of the proposed cooperative tracking model. Following the recursive Bayesian framework, the involved prediction step (5) and correction step (6) are derived to achieve joint estimation of the system state and the noise parameter.

### 4.1. Prediction

We assume the system state $\;x_{k}\;$ and the noise parameter ${\;\Sigma }_{k}^{2}$ are independent to each other given their previous estimation. Therefore, we have

(15) $$p\left(x_{k},\Sigma_{k}^{2}|x_{k-1},\Sigma_{k-1}^{2}\right)=\;p(x_{k}|x_{k-1})p(\Sigma_{k}^{2}|\Sigma_{k-1}^{2}),$$

Substituting (7) and (15) into (5) yields

(16) $$p\left(x_{k},\Sigma_{k}^{2}|y_{1:k-1}\right)=p\left(x_{k}|y_{1:k-1}\right)p\left(\Sigma_{k}^{2}|y_{1:k-1}\right),$$

where we have

(17) $$\{\begin{array}{c} p\left(x_{k}|y_{1:k-1}\right)=\int^{\;}p\left(x_{k}|x_{k-1}\right)p\left(x_{k-1}|y_{1:k-1}\right)dx_{k-1}\;\\ p\left(\Sigma_{k}^{2}|y_{1:k-1}\right)=\int p(\Sigma_{k}^{2}|\Sigma_{k-1}^{2})p\left(\Sigma_{k-1}^{2}|y_{1:k-1}\right)d\Sigma_{k-1}^{2} \end{array},$$

Considering the state evolution model (1) and the posterior distribution of the system state at time step k−1 in (8), we can have

(18) $$p\left(x_{k}|y_{1:k-1}\right)=N\left(x_{k};{\hat{x}}_{k|k-1},P_{k|k-1}\right),$$

where ${\hat{x}}_{k|k-1}$ and $P_{k|k-1}$ are given by the Kalman filter prediction equations

(19) $$\{\begin{array}{c} {\hat{x}}_{k|k-1}=F_{k}{\hat{x}}_{k-1|k-1}\;\;\;\;\;\;\;\\ P_{k|k-1}=F_{k}P_{k-1|k-1}F_{k}^{T}+Q \end{array},$$

For the prediction of the localization noise covariance, it is difficult to specify the evolution model which has the desirable conjugative property. In order to implement recursive estimation and consider the time-varying characteristics of unknown localization noise covariance, a heuristic model [31] is assumed such that

(20) $$p\left(\Sigma_{k}^{2}|y_{1:k-1}\right)=\prod_{t=1}^{m_{2}}IG\left(\sigma_{k|k-1,t}^{2};\alpha_{k|k-1,t},\beta_{k|k-1,t}\right),$$

where the involved scale and the shape parameters are given by

(21) $$\{\begin{array}{c} \alpha_{k|k-1,t}=\rho \alpha_{k-1|k-1,t}\\ \beta_{k|k-1,t}=\rho \beta_{k-1|k-1,t} \end{array},$$

for $\;t=1,2,\ldots ,m_{2}$. Here, ρ (0<ρ≤1) is called the forgetting factor reflecting the fluctuation characteristics of noise statistics. Finally, substituting (18) and (20) into (16), we obtain the prediction distribution of the system state and the localization noise covariance as follows

(22) $$p\left(x_{k},\Sigma_{k}^{2}|y_{1:k-1}\right)=N(x_{k};{\hat{x}}_{k|k-1},P_{k|k-1})\times \prod_{t=1}^{m_{2}}IG\left(\sigma_{k|k-1,t}^{2};\alpha_{k|k-1,t},\beta_{k|k-1,t}\right),$$

### 4.2. Correction

It can be seen from the correction equation (6) that solving the joint posterior distribution $p\left(x_{k},\Sigma_{k}^{2}|y_{1:k}\right)$ of the system state and the localization noise covariance involves multiple integrals and thus is difficult to calculate directly. Therefore, following the VB inference principle described in Section 3, we construct the variational posterior distribution $\;q\left(x_{k},\Sigma_{k}^{2}\right),$ which approximates the true posterior distribution $p\left(x_{k},\Sigma_{k}^{2}|y_{1:k}\right)$. Therefore, we have

(23) $$p\left(x_{k},\Sigma_{k}^{2}|y_{1:k}\right)\approx q\left(x_{k},\Sigma_{k}^{2}\right)=Q_{x}\left(x_{k}\right)Q_{\Sigma }\left(\Sigma_{k}^{2}\right),$$

where $Q_{x}\left(x_{k}\right)$ and $Q_{\Sigma }\left(\Sigma_{k}^{2}\right)$ are the approximate probability densities of the unknown $x_{k}$ and $\Sigma_{k}^{2}$. The KL divergence between the separable approximate distribution and the true posterior distribution is

(24) $$\begin{array}{c} KL\left(Q_{x}\left(x_{k}\right)Q_{\Sigma }\left(\Sigma_{k}^{2}\right)||p\left(x_{k},\Sigma_{k}^{2}|y_{1:k}\right)\right)\\ =\int Q_{x}\left(x_{k}\right)Q_{\Sigma }\left(\Sigma_{k}^{2}\right)\log\left(\frac{Q_{x}\left(x_{k}\right)Q_{\Sigma }\left(\Sigma_{k}^{2}\right)}{p\left(x_{k},\Sigma_{k}^{2}|y_{1:k}\right)}\right)dx_{k}d\Sigma_{k}^{2}, \end{array}$$

According to equation (14), the logarithmic expression of the approximate distribution of $x_{k}\;$ and ${\;\Sigma }_{k}^{2}\;$ is given by

(25) $$\{\begin{array}{c} logQ_{x}\left(x_{k}\right)\propto \int \log p\left(y_{k},x_{k},\Sigma_{k}^{2}|y_{1:k-1}\right)Q_{\Sigma }\left(\Sigma_{k}^{2}\right)d\Sigma_{k}^{2}\\ logQ_{\Sigma }\left(\Sigma_{k}^{2}\right)\propto \int \log p\left(y_{k},x_{k},\Sigma_{k}^{2}|y_{1:k-1}\right)Q_{x}\left(x_{k}\right)dx_{k} \end{array},$$

Furthermore, according to the graphical model shown in Figure 2, the log joint posterior distribution of $y_{k}^{1}$, $y_{k}^{2}$, $x_{k}$ and $\Sigma_{k}^{2}$ can be expressed as

(26) $$\begin{array}{c} \log p\left(y_{k}^{1},y_{k}^{2},x_{k},\Sigma_{k}^{2}|y_{1:k-1}\right)=\log N\left(y_{k}^{1};h\left(x_{k}\right),\Sigma_{k}^{1}\right)+\log N\left(y_{k}^{2};H_{k}x_{k},\Sigma_{k}^{2}\right)\\ +\log N\left(x_{k};{\hat{x}}_{k|k-1},P_{k|k-1}\right)+\log\prod_{t=1}^{m_{2}}IG\left(\sigma_{k|k-1,t}^{2};\alpha_{k|k-1,t},\beta_{k|k-1,t}\right), \end{array}$$

Now, we focus on the derivation of the variational posterior distribution of $\;x_{k\;}$. Firstly, as can be seen from (3), the measurement equation $h\left(x_{k}\right)\;$ is nonlinear in the system state $x_{k}$, thus preventing the recursive estimation. To deal with this problem, we follow the linearization of extended Kalman filter (EKF) and expand the nonlinear function into a Taylor series. By omitting the terms higher than order two, we can obtain the linearized model of the nonlinear system. Specifically, let the Jacobian matrix of the measurement equation $h\left(x_{k}\right)\;$ at the predicted system state $\;{\hat{x}}_{k|k-1}\;$ be

(27) $$JH_{k}={\frac{\partial h\left(x\right)}{\partial x}|}_{x={\hat{x}}_{k|k-1}},$$

For convenience, the observation and the measurement equation can be combined as follows

(28) $$\{\begin{array}{c} y_{k}={\left[y_{k}^{1},y_{k}^{2}\right]}^{T}\\ {\bar{H}}_{k}={\left[JH_{k},H\right]}^{T} \end{array},$$

Then, by substituting (26) and (28) into (25), we can have

(29) $$\begin{array}{c} \log Q_{x}\left(x_{k}\right)\propto -\frac{1}{2}{\left(y_{k}-{\bar{H}}_{k}{\hat{x}}_{k|k-1}\right)}^{T}{\left(\Sigma_{k}\right)}^{-1}\left(y_{k}-{\bar{H}}_{k}{\hat{x}}_{k|k-1}\right)\\ -\frac{1}{2}{\left(x_{k}-{\hat{x}}_{k|k-1}\right)}^{T}{\left(P_{k|k-1}\right)}^{-1}\left(x_{k}-{\hat{x}}_{k|k-1}\right), \end{array}$$

where $\Sigma_{k}=\mathrm{diag}\left(\Sigma_{k}^{1},{\langle \Sigma_{k}^{2}\rangle }_{\Sigma }\right)$ with ${\langle \Sigma_{k}^{2}\rangle }_{\Sigma }=\int \Sigma_{k}^{2}Q_{\Sigma }\left(\Sigma_{k}^{2}\right)d\Sigma_{k}^{2}$, representing the expectation of $\Sigma_{k}^{2}$ with respect to the variational posterior distribution $Q_{\Sigma }\left(\Sigma_{k}^{2}\right)$. According to (29), we immediately find that the variational posterior distribution of $\;x_{k\;}$ is Gaussian $Q_{x}\left(x_{k}\right)=N\left(x_{k};{\hat{x}}_{k|k},P_{k|k}\right),$ where the parameters $\;{\hat{x}}_{k|k}\;$ and $\;P_{k|k}$ are given by

(30) $$S_{k}={\bar{H}}_{k}P_{k|k-1}{\bar{H}}_{k}^{T}+\Sigma_{k},$$

(31) $$K_{k}=P_{k|k-1}{\bar{H}}_{k}^{T}{\left(S_{k}\right)}^{-1},$$

(32) $${\hat{x}}_{k|k}={\hat{x}}_{k|k-1}+K_{k}\left(y_{k}-{\bar{H}}_{k}{\hat{x}}_{k|k-1}\right),$$

(33) $$P_{k|k}=\left(I-K_{k}{\bar{H}}_{k}\right)P_{k|k-1},$$

In order to derive the variational posterior distribution of $\Sigma_{k}^{2}$, we substitute (26) into (25) and obtain

(34) $$\begin{array}{c} \log Q_{\Sigma }\left(\Sigma_{k}^{2}\right)\propto -\sum_{t=1}^{m_{2}}\left(\alpha_{k|k-1,t}+\frac{3}{2}\right)\log\sigma_{k|k,t}^{2}-\sum_{t=1}^{m_{2}}\frac{1}{\sigma_{k|k,t}^{2}}\{\beta_{k|k-1,t}+\\ \frac{1}{2}{\left(HP_{k|k}H^{T}+\left(y_{k}^{2}-H{\hat{x}}_{k|k}\right){\left(y_{k}^{2}-H{\hat{x}}_{k|k}\right)}^{T}\right)}_{tt}\} \end{array},$$

As a result, we find that $\Sigma_{k}^{2}$ follows inverse-Gamma distribution $Q_{\Sigma }\left(\Sigma_{k}^{2}\right)=\prod_{t=1}^{m_{2}}IG\left(\sigma_{k|k,t}^{2};\alpha_{k|k,t},\beta_{k|k,t}\right)$, with the parameters $\alpha_{k|k,t},\beta_{k|k,t}$ $\left(t=1,2,\ldots ,m_{2}\right)$ given by

(35) $$\alpha_{k|k,t}=\alpha_{k|k-1,t}+\frac{1}{2},$$

(36) $$\beta_{k|k,t}=\beta_{k|k-1,t}+\frac{1}{2}{\left(HP_{k|k}H^{T}+\left(y_{k}^{2}-H{\hat{x}}_{k|k}\right){\left(y_{k}^{2}-H{\hat{x}}_{k|k}\right)}^{T}\right)}_{tt},$$

(37) $$\langle \Sigma_{k}^{2}\rangle =\mathrm{diag}\left(\frac{\beta_{k|k,1}}{\alpha_{k|k,1}},\ldots ,\frac{\beta_{k|k,m_{2}}}{\alpha_{k|k,m_{2}}}\right),$$

Considering the correction equations of $Q_{x}\left(x_{k}\right)$ and $Q_{\Sigma }\left(\Sigma_{k}^{2}\right)$ are dependent on each other, the posterior distribution parameters need to be calculated alternatively such that more accurate approximation can be achieved. Considering the calculation cost, we present two termination conditions

**Condition 1:** If the state change between two adjacent iterations is less than the threshold, then the VB iteration will converge. The specific formula is

(38) $$\frac{\|{\hat{x}}_{k|k}^{i+1}-{\hat{x}}_{k|k}^{i}{\|}_{2}}{\|{\hat{x}}_{k|k}^{i}{\|}_{2}}<\varepsilon ,$$

where ${\left\|\cdot \right\|}_{2}$ is the Euclidean norm.

**Condition 2:** If the number of VB iterations reaches the predetermined maximum number of iterations c, the VB iteration will also be terminated.

### 4.3. Algorithm

In summary, the proposed cooperative tracking algorithm under time-varying localization noise can be described as follows (Algorithm 1).
**Algorithm 1 Multi-Vehicle Cooperative Target Tracking****Initialization:**${\hat{x}}_{1|1}$,$\;P_{1|1}$,${\left\{\alpha_{1|1,t},\beta_{1|1,t}\right\}}_{t=1}^{m_{2}}$**Time outer loop**
For k=2,3,⋯ **Prediction:**
 ${\hat{x}}_{k|k-1}=F_{k}{\hat{x}}_{k-1|k-1}$ $P_{k|k-1}=F_{k}P_{k-1|k-1}F_{k}^{T}+Q$ $\alpha_{k|k-1,t}=\rho \alpha_{k-1|k-1,t}\;\;t=1,2,\ldots ,m_{2}$ $\beta_{k|k-1,t}=\rho \beta_{k-1|k-1,t}\;\;t=1,2,\ldots ,m_{2}$ **Correction:** $JH_{k}={\frac{\partial h\left(x\right)}{\partial x}|}_{x={\hat{x}}_{k|k-1}}$ ${\bar{H}}_{k}={\left[JH_{k},H\right]}^{T}$ $y_{k}={\left[y_{k}^{1},y_{k}^{2}\right]}^{T}$ $\alpha_{k|k,t}^{0}=\alpha_{k|k-1,t}$ $\beta_{k|k,t}^{0}=\beta_{k|k-1,t}$ **Variational inner loop** For i=0,1,2,⋯  ${\langle \Sigma_{k}^{2}\rangle }^{i+1}=\mathrm{diag}\left(\frac{\beta_{k|k,1}^{i}}{\alpha_{k|k,1}^{i}},\ldots ,\frac{\beta_{k|k,m_{2}}^{i}}{\alpha_{k|k,m_{2}}^{i}}\right)$  $\Sigma_{k}^{i+1}=\mathrm{diag}\left(\Sigma_{k}^{1},{\langle \Sigma_{k}^{2}\rangle }^{i+1}\right)$  $S_{k}^{i+1}={\bar{H}}_{k}P_{k|k-1}{\bar{H}}_{k}^{T}+\Sigma_{k}^{i+1}$  $K_{k}^{i+1}=P_{k|k-1}{\bar{H}}_{k}^{T}{\left(S_{k}^{i+1}\right)}^{-1}$  ${\hat{x}}_{k|k}^{i+1}={\hat{x}}_{k|k-1}+K_{k}^{i+1}\left(y_{k}-{\bar{H}}_{k}{\hat{x}}_{k|k-1}\right)$  $P_{k|k}^{i+1}=\left(I-K_{k}^{i+1}{\bar{H}}_{k}\right)P_{k|k-1}$  $\alpha_{k|k,t}^{i+1}=\alpha_{k|k-1,t}+\frac{1}{2}\;\;t=1,2,\ldots ,m_{2}$  $\beta_{k|k,t}^{i+1}=\beta_{k|k-1,t}+\frac{1}{2}{\left(HP_{k|k}^{i+1}H^{T}+\left(y_{k}^{2}-H{\hat{x}}_{k|k}^{i+1}\right){\left(y_{k}^{2}-H{\hat{x}}_{k|k}^{i+1}\right)}^{T}\right)}_{tt}\;\;t=1,2,\ldots ,m_{2}$  **Convergence judgment**  Condition 1: $\frac{\|{\hat{x}}_{k|k}^{i+1}-{\hat{x}}_{k|k}^{i}{\|}_{2}}{\|{\hat{x}}_{k|k}^{i}{\|}_{2}}<\varepsilon$  Condition 2: i>c **End the inner loop** ${\hat{x}}_{k|k}={\hat{x}}_{k|k}^{I+1},P_{k|k}=P_{k|k}^{I+1}$, I is the number of VB iteration**End the outer loop****Outputs:**$\;{\left\{{\hat{x}}_{k|k},P_{k|k}\right\}}_{k=2,3,\cdots }$, 

## 5. Simulation and Discussion

### 5.1. Simulation Scene Configuration

Assume that the target and CV move in a 2-D plane with the constant velocity model. The position of target can be observed simultaneously by HV and CV. In addition, the position of CV can be obtained by HV through inter-vehicle communication. Taking the coordinate frame of HV as reference frame, the initial states of the target and the cooperative vehicle are ${\left[30,15,1,1\right]}^{T}$ and ${\left[20,20,2,2\right]}^{T}$, respectively. The state transition matrix $F_{k}$ in (1) is given by

$$F_{k}=\left[\begin{array}{ccc} F^{1} & & \\ & F^{1} & \\ & & F^{2} \end{array}\right]$$

where $F^{1}=\left[\begin{array}{cccc} 1 & 0 & ∆t & 0\\ 0 & 1 & 0 & ∆t\\ 0 & 0 & 1 & 0\\ 0 & 0 & 0 & 1 \end{array}\right]$, $F^{2}=\left[\begin{array}{cc} 1 & ∆t\\ 0 & 1 \end{array}\right]$, the sampling period ∆t=0.1. The total number of time steps is 400 and thus the actual simulation time is 40 s. The heading angle of the cooperative vehicle is simulated according to the following equation

$$\theta_{k}=\tan^{-1}\frac{{\dot{p}}_{y,k}^{13}}{{\dot{p}}_{x,k}^{13}}+\frac{5}{100}\sin\frac{k}{100}$$

The process noise $\;w_{k}\;$ in (1) is assumed to be zero-mean white Gaussian noise with covariance matrix $Q=\mathrm{diag}\left(Q_{1},Q_{2}\right)$, where $Q_{1}=I_{2}⨂0.01^{2}GG^{T}$, is the 2-D identity matrix, ⨂  is the Kronecker product. The expression of G and $Q_{2}$ is given below [36]

$$G=\left[\begin{array}{cccc} \frac{∆t^{3}}{3} & 0 & \frac{∆t^{2}}{2} & 0\\ 0 & \frac{∆t^{3}}{3} & 0 & \frac{∆t^{2}}{2}\\ \frac{∆t^{2}}{2} & 0 & ∆t & 0\\ 0 & \frac{∆t^{2}}{2} & 0 & ∆t \end{array}\right],\;Q_{2}=\left[\begin{array}{cc} \frac{∆t^{3}}{3} & \frac{∆t^{2}}{2}\\ \frac{∆t^{2}}{2} & ∆t \end{array}\right]$$

**Figure 3:**
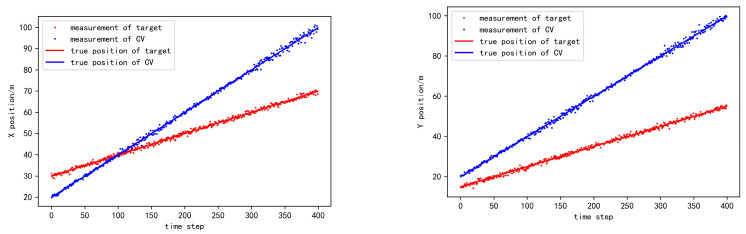
The position observation of the target and the cooperative vehicle in HV coordinate frame.

**Figure 4:**
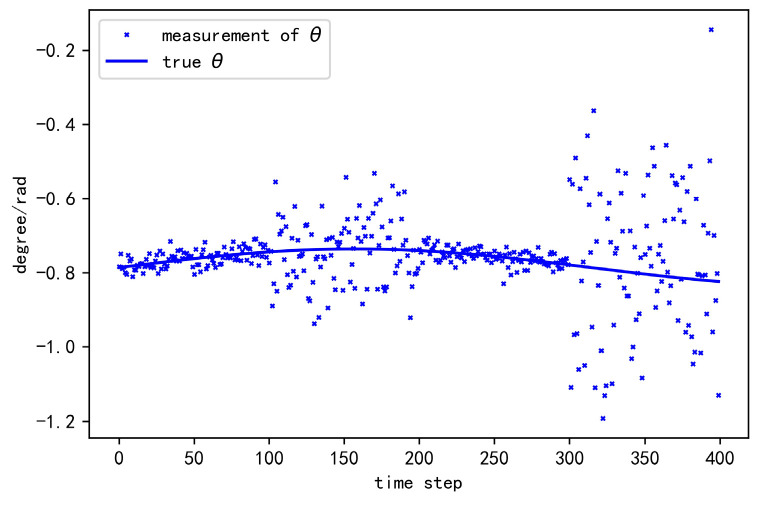
The heading angle observation of the cooperative vehicle in HV coordinate frame.

**Figure 5:**
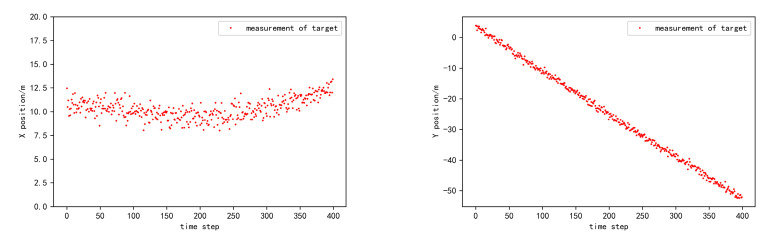
The position observation of the target in CV coordinate frame.

Suppose that the observation noises $v_{k}^{1}$ and $v_{k}^{2}$ in (2) obey Gaussian distribution with zero mean and covariance matrix. Specifically, for the on-board sensor measurement noise $v_{k}^{1}$, we let the corresponding covariance $\Sigma_{k}^{1}=\mathrm{diag}\left(0.5,0.5,0.5.0.5\right)$. For the localization noise $v_{k}^{2}$, we simulate the time-varying covariance $\Sigma_{k}^{2}$ at time step k as follows

$$\Sigma_{k}^{2}=\{\begin{array}{ccc} \Sigma , & & \mathrm{if}\;k\in \left[1,100\right]\;\\ 5\Sigma , & & \mathrm{if}\;k\in \left[101,200\right]\\ \Sigma , & & \mathrm{if}\;k\in \left[201,300\right]\\ 10\Sigma , & & \mathrm{if}\;k\in \left[301,400\right] \end{array}$$

where $\;\Sigma =\mathrm{diag}\left(\sigma^{2},\sigma^{2},0.1\times \sigma^{2}\right)$. In the simulation, we let $\sigma^{2}$ vary in the set {0.1,0.2,0.3,0.4,0.5}, so as to investigate the tracking performance under different levels of localization uncertainty. Take $\sigma^{2}=0.2$ as an instance. Figure 3 shows the observed position of the target and CV in the coordinate frame of HV. Figure 4 shows the observed heading angle of CV in the coordinate frame of HV. Figure 5 shows the observed position of the target in the coordinate frame of CV.

With the above simulated scene, computer experiments are carried out to evaluate the performance of our proposed algorithm, termed as variational Bayesian inference-cooperative tracking (VBI-CT). For comparison, we include three related methods: Kalman filter (KF), EKF-cooperative tracking/static (EKF-CT/S) and EKF-cooperative tracking/dynamic (EKF-CT/D). For KF, we estimate the state of target based solely on HV without cooperation. For EKF-CT/S, we assume the potential localization noise covariance $\Sigma_{k}^{2}$ is static and set to Σ, and thus the state of target and CV (i.e., (1) and (2)) can be recursively estimated by EKF. For EKF-CT/D, it is similar to EKF-CT/S, except that we assume the perfect knowledge regarding the dynamic localization noise is provided (i.e., the exact value of $\Sigma_{k}^{2}$ at each time step is known). For all the algorithms, the initial state of the target and CV is set as the corresponding position observation while the velocity is simply set as zero since no prior information is available. For VBI-CT, there are some parameters need to set before online estimation. Specifically, the convergence threshold of VB iteration $\varepsilon =5\times 10^{-6}$, the forgetting factor ρ=0.7. the maximum number of iterations c=10, the initial value for the localization noise parameter α and β is simply set as 1.0.

To eliminate the influence of randomness, we perform a total of 100 Monte Carlo (MC) runs to compare KF, EKF-CT/S, EKF-CT/D, and VBI-CT. To evaluate the tracking performance of different algorithms, we adopt the root mean squared error (RMSE) and averaged root mean squared error (ARMSE), defined as

$$RMSE\left(k\right)=\sqrt{\frac{1}{M}\overset{M}{\underset{m=1}{\sum }}\left({\left(p_{x,k}-{\hat{p}}_{x,k}^{m}\right)}^{2}+{\left(p_{y,k}-{\hat{p}}_{y,k}^{m}\right)}^{2}\right)}$$

$$ARMSE_{k}=\frac{1}{K}\overset{K}{\underset{k=1}{\sum }}RMSE\left(k\right)$$

where $\left(p_{x,k},p_{y,k}\right)$ denotes the true target position at time step  k, while $\;({\hat{p}}_{x,k}^{m},{\hat{p}}_{y,k}^{m})\;$ is the corresponding estimation at the mth  MC run. As can be seen, ARMSE is the average RMSE across the whole simulation time.

### 5.2. Tracking Performance under Time-Varying Localization Noise

**Figure 6:**
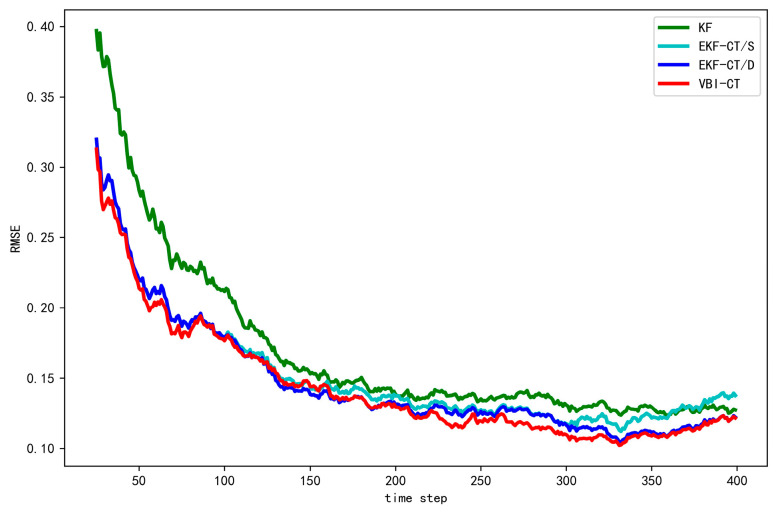
Variation curves of tracking errors obtained by different algorithms when $\sigma^{2}=0.2$

As mentioned above, we have performed a total of 100 MC runs to calculate the tracking error of four algorithms. The experimental results obtained by KF, EKF-CT/S, EKF-CT/D, and VBI-CT, when the localization noise covariance $\sigma^{2}$ increases from 0.1 to 0.5, are shown in Table 1, Table 2, Table 3, Table 4 and Table 5, respectively. Figure 6 shows the RMSE curve of different algorithms when $\sigma^{2}=0.2$.

By analyzing the above experimental results, we find that the overall performance of KF is the worst among the three algorithms. The reason for the poor performance is that the target state is estimated exclusively based on the observations of HV without considering the information from CV. EKF-CT/S outperforms KF when the localization noise covariance $\sigma^{2}$ is small, indicating the information from CV has been successfully integrated with the local observation of HV. However, with the increase of $\sigma^{2}$, the performance of EKF-CT/S degenerates. This is because EKF-CT/S cannot adjust the localization noise covariance automatically, thus lacking adaptation to the dynamic environment. EKF-CT/D and VBI-CT work better than the other two methods, since they can not only integrate the information from CV but also take into account the dynamic variation of the localization noise uncertainty. We observe from the results that the performance of VBI-CT is very close to that of EKF-CT/D, which assumes the exact time-varying localization noise is known. As a result, the application of EKF-CT/D is restricted in many real applications where the uncertainty of the localization error is difficult or even impossible to obtain. Our proposed VBI-CT, on the other hand, can estimate such uncertainty automatically based on the observed measurements, thus verifying the effectiveness of VBI-CT in the dynamic environment. In addition, we notice that, with the increase of the localization noise variance, the tracking error of VBI-CT increases correspondingly because of the position uncertainty of CV. Nevertheless, in comparison with KF, we can find the tracking error of VBI-CT can reduce about 18.15% to 9.5%, depending on the uncertainty level of the localization noise.

### 5.3. Run Time Comparison

The averaged execution time of all algorithms across 100 MC runs is shown in Table 6. It should be noticed that the execution time includes the calculation in 400 time steps. In terms of execution time, compared with the other algorithms, KF consumes the least time because it only needs to estimate the state of target, thus leading to very small state space. The speed of EKF-CT/S and EKF-CT/D is slower than that of KF because they both have to estimate the joint state of target and CV, thus leading to larger state space. Finally, our proposed VBI-CT algorithm takes the longest time, because it needs to estimate more parameters and iterate the VB inference until convergence. Nevertheless, the time consumed in each time step is still very small (~5 ms).

### 5.4. Influence of Parameters

**Figure 7:**
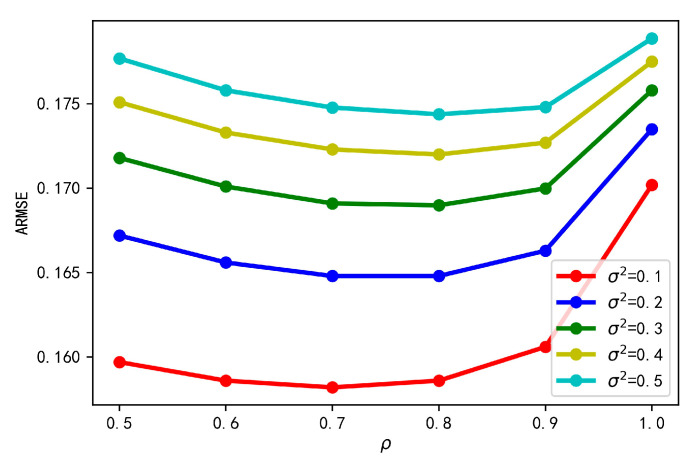
Tracking error of the proposed algorithm with different forgetting factor.

For the proposed VBI-CT, the forgetting factor 0<ρ≤1 is used to initialize the prior distribution of the localization noise at each time step, thus will affect the subsequent VB inference procedure. A large ρ should be chosen when the variation of the localization noise over time is slow, wheras a small ρ is more preferred if the localization noise changes fast. Hence, we analyze the variation of tracking error with respect to different values of ρ. We increase the value from 0.5 to 1.0 with step 0.1. The resulting variation curve of ARMSE is shown in Figure 7. It can be seen that the tracking error gradually decreases with the increased value of ρ at the initial stage. However, when ρ is too large, the tracking error will increase due to the excessive self-pruning of the parameters by the VB algorithm [32]. Therefore, in the simulation, we set ρ as 0.7.

**Figure 8:**
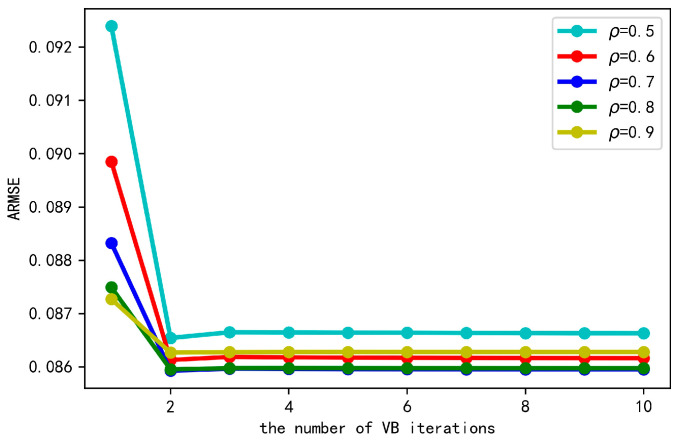
Tracking error of the proposed algorithm under different numbers of variational Bayesian (VB) iterations.

The number of iterations in VB inference is an important factor affecting the tracking behavior. Generally, with the increase of iterations, variational distribution can approximate the true posterior distribution more precisely, thus resulting in better tracking performance. However, too many iterations will bring about heavier computational burden and reduce the tracking speed. Therefore, we investigate how the tracking error varies with the increased number of VB iterations. The result is shown in Figure 8. where the number of VB iterations increases from 1 to 10. We also show different variation curves under different values of forgetting factor. As can be seen, the tracking error decreases rapidly during the first several iterations and reaches the convergence after about 3 iterations. Therefore, the proposed cooperative tracking method shows fast convergence speed with respect to the number of VB iterations.

**Figure 9:**
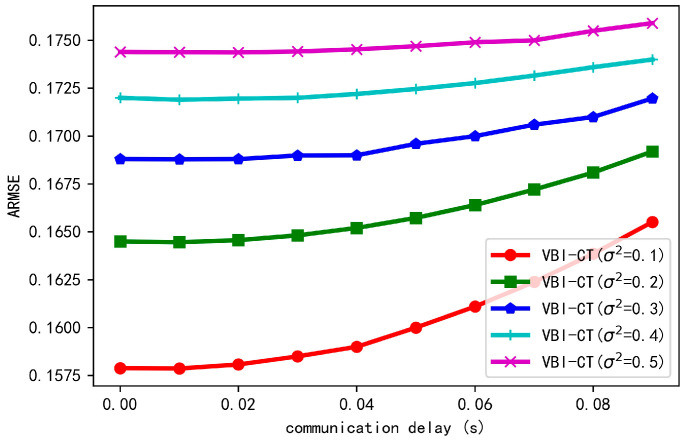
Tracking error of the proposed algorithm under different communication delays.

In the above experiments, we assume the information from CV can be immediately transmitted to HV without delays. In such a case, the transmitted information and the local perception of HV can be temporally aligned. However, in real applications, due to the communication band or the large number of vehicles involved in cooperation, communication delay is an important factor influencing the performance of cooperative tracking. Therefore, we study the tracking error under different communication delays. We increase the delay from 0.00 to 0.09 s with the step 0.01 and show the results in Figure 9. As we can see, with the increase of communication delay, the error of cooperative tracking gradually increases and the impact of delay on cooperative tracking is larger when the localization uncertainty is small.

### 5.5. Tracking Performance under Stationary Localization Noise

**Figure 10:**
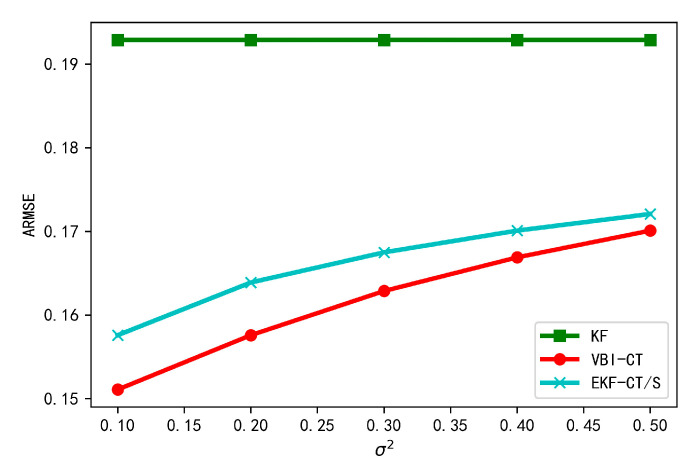
Tracking error under stationary localization noise.

In this experiment, we assess the tracking performance of different approaches when the localization noise is stationary so as to evaluate the applicability of our proposed algorithm. In this case, EKF-CT/D will reduce to EKF-CT/S since the covariance of localization noise is constant. We gradually increase the value of $\sigma^{2}$ from 0.1 to 0.5 and show the corresponding results in Figure 10. As can be seen, VBI-CT and EKF-CT/S algorithms still outperform KF in terms of state estimation, which further verifies the advantages of cooperative tracking over non-cooperative tracking. We also observe that, as the relative localization noise increases, the error of cooperative tracking algorithms increases gradually, thus emphasizing the importance of relative localization in the cooperative scenario.

## 6. Conclusions

In this paper, a cooperative target tracking algorithm is developed for the integration of the information from multiple vehicles. This method attempts to jointly estimate the state of target and CV as well as the localization noise parameter modeled by inverse-Gamma distribution. The method is formulated in the recursive Bayesian framework, where the posterior distribution of the unknown variables is dealt with variational Bayesian inference. The performance of the proposed method is verified using computer simulation. The results through 100 Monte Carlo runs show that cooperative tracking can effectively reduce the tracking error even with time-varying localization uncertainty.

## Figures and Tables

**Table 1:** Comparison of tracking error of different algorithms with $\sigma^{2}=0.1$.

Time Period	KF	EKF-CT/S	EKF-CT/D	VBI-CT
[1‒100]	0.3456	0.2679	0.2679	0.2739
[100‒200]	0.1622	0.1419	0.1411	0.1390
[200‒300]	0.1370	0.1233	0.1228	0.1122
[300‒400]	0.1281	0.1184	0.1116	0.1073
[1‒400]	0.1928	0.1626	0.1606	0.1578

**Table 2:** Comparison of tracking error of different algorithms with $\sigma^{2}=0.2$.

Time Period	KF	EKF-CT/S	EKF-CT/D	VBI-CT
[1‒100]	0.3456	0.2816	0.2816	0.2810
[100‒200]	0.1622	0.1509	0.1461	0.1475
[200‒300]	0.1370	0.1280	0.1258	0.1195
[300‒400]	0.1281	0.1249	0.1139	0.1111
[1‒400]	0.1928	0.1711	0.1666	0.1645

**Table 3:** Comparison of tracking error of different algorithms with $\sigma^{2}=0.3$.

Time Period	KF	EKF-CT/S	EKF-CT/D	VBI-CT
[1‒100]	0.3456	0.2900	0.2900	0.2872
[100‒200]	0.1622	0.1561	0.1490	0.1516
[200‒300]	0.1370	0.1307	0.1274	0.1237
[300‒400]	0.1281	0.1286	0.1154	0.1138
[1‒400]	0.1928	0.1761	0.1701	0.1688

**Table 4:** Comparison of tracking error of different algorithms with $\sigma^{2}=0.4$.

Time Period	KF	EKF-CT/S	EKF-CT/D	VBI-CT
[1‒100]	0.3456	0.2958	0.2958	0.2926
[100‒200]	0.1622	0.1596	0.1509	0.1541
[200‒300]	0.1370	0.1326	0.1285	0.1266
[300‒400]	0.1281	0.1310	0.1164	0.1158
[1‒400]	0.1928	0.1795	0.1726	0.1720

**Table 5:** Comparison of tracking error of different algorithms with $\sigma^{2}=0.5$.

Time Period	KF	EKF-CT/S	EKF-CT/D	VBI-CT
[1‒100]	0.3456	0.3001	0.3001	0.2972
[100‒200]	0.1622	0.1622	0.1523	0.1558
[200‒300]	0.1370	0.1340	0.1294	0.1286
[300‒400]	0.1281	0.1325	0.1173	0.1172
[1‒400]	0.1928	0.1819	0.1744	0.1744

**Table 6:** Total execution time (s) of different algorithms.

$\sigma^{2}$	KF	EKF-CT/S	EKF-CT/D	VBI-CT
0.1	0.0192	0.0371	0.0378	0.1803
0.2	0.0202	0.0380	0.0388	0.1880
0.3	0.0252	0.0477	0.0489	0.2331
0.4	0.0205	0.0390	0.0395	0.1894
0.5	0.0227	0.0462	0.0469	0.2197
